# Amelioration effects of the soybean lecithin–gallic acid complex on iron-overload-induced oxidative stress and liver damage in C57BL/6J mice

**DOI:** 10.1080/13880209.2022.2151632

**Published:** 2022-12-27

**Authors:** Caihong Wu, Wenxin Zhang, Feifei Yan, Wenwen Dai, Fang Fang, Yanli Gao, Weiwei Cui

**Affiliations:** a Department of Nutrition and Food Hygiene, School of Public Health, Jilin University, Changchun, China; b Department of Pathogenobiology, Jilin University Mycology Research Center, College of Basic Medical Sciences, Jilin University, Changchun, China; c Department of Pediatric Ultrosonic, The First Hospital of Jilin University, Changchun, China

**Keywords:** Phenolic acid, excessive iron, antioxidant activity, hepatic damage

## Abstract

**Context:**

Gallic acid (GA) and lecithin showed important roles in antioxidant and drug delivery, respectively. A complex synthesized from GA and soybean lecithin (SL-GAC), significantly improved bioavailability of GA and pharmacological activities. However, the antioxidant activity of SL-GAC and its effect on iron-overload-induced liver injury remains unexplored.

**Objective:**

This study investigates the antioxidant properties of SL-GAC *in vitro* and in mice, and its remediating effects against liver injury by iron-overloaded.

**Materials and methods:**

*In vitro*, free radical scavenging activity, lipid peroxidation inhibition, and ferric reducing power of SL-GAC were measured by absorbance photometry. *In vivo*, C57BL/6J mice were randomized into 4 groups: control, iron-overloaded, iron-overloaded + deferoxamine, and iron-overloaded + SL-GAC. Treatments with deferoxamine (150 mg/kg/intraperitioneally) and SL-GAC (200 mg/kg/orally) were given to the desired groups for 12 weeks, daily. Iron levels, oxidative stress, and biochemical parameters were determined by histopathological examination and molecular biological techniques.

**Results:**

*In vitro*, SL-GAC showed DPPH and ABTS free radicals scavenging activity with IC_50_ values equal to 24.92 and 128.36 μg/mL, respectively. In C57BL/6J mice, SL-GAC significantly reduced the levels of serum iron (22.82%), liver iron (50.29%), aspartate transaminase (25.97%), alanine transaminase (38.07%), gamma glutamyl transferase (42.11%), malondialdehyde (19.82%), total cholesterol (45.96%), triglyceride (34.90%), ferritin light chain (18.51%) and transferrin receptor (27.39%), while up-regulated the levels of superoxide dismutase (24.69%), and glutathione (11.91%).

**Conclusions:**

These findings encourage the use of SL-GAC to treat liver injury induced by iron-overloaded. Further *in vivo* and *in vitro* studies are needed to validate its potential in clinical medicine.

## Introduction

Reactive oxygen species (ROS) are typical by-products of cellular metabolism, playing a role as secondary messengers and influencing different normal physiological functions of the body (Villalpando-Rodriguez and Gibson 2021). Increased levels of ROS in living cells can damage DNA, RNA, proteins, and healthy cells, resulting in the development and progression of several serious diseases (Srinivas et al. 2019). Iron is an essential micronutrient, playing a crucial role in many biological processes including oxygen transport and storage, oxidative phosphorylation, and the catalysis of many metabolic redox reactions (Coffey and Ganz 2017), as components of oxygen transport proteins (haemoglobin and myoglobin) and of numerous metabolic and redox enzymes (Sung et al. 2019). Iron is required for a number of diverse cellular functions including DNA synthesis, ATP generation, and cellular proliferation (Puig et al. 2017). The richest sources of iron in the diet are lean meat and seafood. Other dietary sources of iron also include nuts, beans, vegetables, and fortified grain products. However, accumulation of iron in tissues and organs is toxic and can cause organ damage and disrupt normal function, particularly in the liver which is the main storage site for iron in body (Zaccone and Gasbarrini 2012). As the body has no active excretion pathways for excess iron, a continuous load of iron exceeding 1–2 mg/day will result in iron overload (Kohgo et al. 2008). Free cellular iron can participate in the Fenton reaction and catalyse the conversion of hydrogen peroxide to highly toxic hydroxyl radicals (Beaufay et al. 2020). Emerging evidence also suggests that oxidative stress, mediated by free radicals and ROS, may have a role in the pathophysiology of iron-induced liver injury (Galaris et al. 2019). Therefore, a bioactive compound or a drug that effectively traps free iron is required to make it unavailable for Fenton reaction, reduce the formation of highly reactive hydroxyl radicals and the peroxidation of unsaturated lipids, and acts as an antioxidant.

Gallic acid (GA), a naturally occurring phenolic acid widely distributed in a variety of foods such as apples, b7lueberries, walnuts, and watercress, is well known as powerful antioxidant (Sanchez-Martin et al. 2022). It has characteristics of the strong antioxidant and free radical scavenging activities, and can protect biological cells, tissues, and organs from damages caused by oxidative stress (Gao et al. 2019). It has been shown to exhibit numerous biological and pharmacological properties, including antiviral, antimicrobial, anti-inflammatory, and antitumor activities in numerous human cancer cell lines (Kahkeshani et al. 2019). However, poor bioavailability of GA is shown because its poor lipid solubility that don’t allow its penetration into cell membranes, which results in an inability to achieve its desired pharmacological function (Ahmed et al. 2018; Hassani et al. 2020). We previously observed that SL-GAC, a complex synthesized from GA and soybean lecithin, significantly increased bioavailability of GA and improved pharmacological activities *in vivo*, and with no effects on the safety for the organism (Wu et al. 2019). However, the detailed knowledge of antioxidant activity of SL-GAC and amelioration of iron-overload-induced oxidative stress and liver injury remains unexplored. Thus, the present study was designed to investigate the *in vitro* antioxidant properties of SL-GAC and its *in vivo* ameliorating effects against iron-overload induced oxidative stress and liver injury in C57BL/6J mice.

## Materials and methods

### Reagents and antibodies

The standard iron chelating drug, deferoxamine (DFO) was obtained from Novartis International AG (Basel, Switzerland). Carbonyl iron, GA, soybean lecithin, ethanol, vitamin C, ferric chloride, ferrous chloride, linoleic acid, 1,1-diphenyl-2-picrylhydrazyl (DPPH), 2, 2-azinobis (3-ethylbenzo-thiozoline-6-sulfonic acid) (ABTS), potassium ferricyanide and 30% hydrogen peroxide were purchased from Sigma Chemical Co. (St. Louis, MO, USA). The assay kits for alanine transaminase (ALT) (C009-2-1), aspartate transaminase (AST) (C010-2-1), γ-glutamyl transferase (γ-GT) (C017-2-1), and superoxide dismutase (SOD) (A001-3-2), glutathione (GSH) (A006-2-1), malondialdehyde (MDA) (A003-1-1), total cholesterol (TC) (A111-1-1), total iron-binding capacity (TIBC) (A040-1-1), triglyceride (TG) (A110-1-1) were procured from Nanjing Jiancheng Biotechnology Co., Ltd. (Nanjing, China). Antibodies against transferrin receptor (TfR1) (ab84036), Ferritin (ab153976), ferritin light chain (FL) (ab109373) and glyceraldehyde-3-phosphate dehydrogenase (GAPDH) (ab198306) were all purchased from Abcam Inc. (Cambridge, MA, USA).

### Preparation of SL-GAC

The SL-GAC, a stable complex of soybean lecithin and gallic acid was prepared according to our previously described method (Wu et al. 2019). The polar ends of gallic acid and lecithin may interact in the SL–GAC, and gallic acid was completely combined with lecithin, and both retained their characteristic chemical structures and did not undergo a chemical reaction. Further, SL–GAC did not cause any clinical signs of toxicity and was safe for the organism.

### DPPH radical scavenging assay

The SL-GAC's ability to scavenge DPPH free radical was determined using the previous method (Shimada et al. 1992). Fresh DPPH stock solution (0.25 mM) was prepared in ethanol on each day of analysis. Stock solutions of SL-GAC was prepared in ethanol at a concentration of 0.08 mg/mL. Then, 7 separate 10 mL volumetric flasks were taken and aliquots of 0, 0.25, 0.5, 1.0, 2.0, 4.0, and 5.0 mL of 0.08 mg/mL solution of SL-GAC were added, respectively, to separate volumetric flasks. Then, 2 mL of freshly prepared DPPH solution (0.25 mM) was added to each of the mentioned 7 volumetric flasks, and the final volume was made up to 10 mL in all the flasks by the addition of ethanol. The mixture was left in the dark at room temperature for 30 min after the reaction mixture was vortexed thoroughly. Absorbance was measured immediately at 517 nm by UV spectrophotometer and the solution containing only anhydrous ethanol served as the blank. Vitamin C standard antioxidant (2.0, 4.0, 8.0, 16.0, 32.0, and 40.0 μg/mL) was used as a reference standard. DPPH free radical scavenging activity was calculated using the following formula:

$$\text{DPPH scavenging activity}\left(\%\right)=\left[\left({\text{A}}_{0}-{\text{A}}_{\text{1}}\right)/{\text{A}}_{0}\right]\times \text{1}00$$

where A_0_ = absorbance of the blank sample, and A_1_ = absorbance of the test sample.

### ABTS radical cation scavenging activity

The ABTS radical cation scavenging activity was determined according to the method of Re et al. (1999). ABTS was dissolved in deionized water to a 7.0 mM concentration. ABTS radical cation (ABTS^•+^) was produced by reacting ABTS^•+^ solution with 140 mM potassium persulfate and allowing the mixture to stand in the dark for 12–16 h at room temperature before use. Subsequently, the ABTS^•+^ solution was diluted in ethanol to obtain an absorbance of approximately 0.51 ± 0.02 at 734 nm. A volume of 0.6 mL of SL-GAC with different concentrations was added to 2.4 mL amount of ABTS^•+^ solution for 10 s, and stood for 6 min in the dark. The reference compound (vitamin C) was used at the same concentration as the SL-GAC. Thereafter, the absorbance of all test tubes was determined at 734 nm. The solution containing only anhydrous ethanol served as the blank and all determinations were carried out in triplicate. The percentage of inhibition of ABTS^•+^ was calculated using the following formula:

$$\text{ABTS radical scavenging activity }\left(\%\right)=\left[\left({\text{A}}_{0}-{\text{A}}_{\text{1}}\right)/{\text{A}}_{0}\right]\times \text{1}00$$

where A_0_ = absorbance of the blank sample, and A_1_ = absorbance of the test sample.

### Inhibition of linoleic acid autoxidation

The ferric thiocyanate method (Ozsoy et al. 2008) was used to determine the inhibition of peroxidation of linoleic acid. Five mL of SL-GA (10 mg/mL) were added to a solution mixture of 2.5% linoleic acid in ethanol (4 mL), anhydrous ethanol (4 mL), and 8 mL of 0.05 M phosphate buffer (pH = 7.0). Total mixture was diluted to 25 mL with distilled water, and the final concentration of SL-GAC was 2.0 mg/mL. The solution was incubated and then placed in an oven at 40 °C in the dark. The degree of oxidation was measured every 24 h by sequentially adding 0.1 mL of the SL-GAC sample solution, 9.7 mL of 75% ethanol, 0.1 mL of 30% ammonium thiocyanate and 0.1 mL of 0.02 M ferrous chloride in 3.5% hydrochloric acid. The mixture was let to stand for 3 min, the peroxide concentration was spectrophotometrically determined by reading the absorbance at 500 nm.

### Hydroxyl radical scavenging assay

Different concentrations of the SL-GAC solution (40, 80, 120 and 160 μg/mL) were added to reagent solution (7.5 mM iron sulphate, 5 M diphenanthrene solution in ethanol, 0.01 M phosphate buffer, 1% H_2_O_2_) and incubated at 37 °C for 1 h. The reaction mixture without sample was used as control. The hydroxyl radical was investigated by monitoring absorbance at 536 nm.

### Ferric reducing antioxidant potential assay

The ferric reducing power of SL-GAC was determined using Ferric reducing antioxidant potential (FRAP) assay. This method is based on the reduction of the ferric ion (Fe^3+^) to the ferrous ion (Fe^2+^) by antioxidants in the sample (Gülçin 2015). The reduction is monitored by measuring the change of absorbance at 700 nm. The working FRAP reagent was prepared daily by mixing 30 mL of 10 mg/mL potassium ferricyanide solution, with 7.5 mL of 1 mg/mL ferric chloride solution, with 30 mL of 0.01 M phosphate buffered solution and with 7.5 mL of 1.2 mM hydrochloric acid. Different volumes of SL-GAC reserve solution (1.6 mg/mL) were mixed with freshly prepared FRAP reagent (5 mL), and deionized water was added to 10 mL. The reaction mixture was incubated for 30 min at room temperature, and the absorbance of the samples was measured at 700 nm. Increased absorbance of the reaction mixture indicated increased reducing power.

### Establishment of iron overload-induced liver injury in mice

The animal study was performed according to the guidelines established by the National Institutes of Health, and with permission from the Animal Experiment Committee of Jilin University (approval number: 20180303). Healthy male C57BL/6J mice (8 weeks old) were purchased from Changsheng Experimental Animal Company (Liaoning, China), and were kept at 23 ± 2 °C under a 12 h dark/light cycle, with a relative humidity of 50–55% and allowed access to normal laboratory pellet diet and water *ad libitum*. After acclimatizing for a week and weighing, mice were randomly divided into four groups: control, model (FE), model + DFO (FED), and SL-GAC groups, with each group containing 10 mice. Except for the control group, which was provided with a regular diet and physiological saline, the other three groups were fed with ordinary diet supplemented with 3% carbonyl iron for 12 weeks to induce iron-overload condition. In the meantime, from week 2, in addition to the same treatment as that of the FE group, mice of FED and SL-GAC groups were intraperitoneally injected with DFO (150 mg/kg body weight), intragastrically administered with SL-GAC (200 mg/kg body weight) every day, respectively. DFO is poorly absorbed from the gastrointestinal tract when taken orally, resulting in a decrease of its bioavailability. Therefore, iron overload-induced liver injury mice were treated with DFO *via* the method of intraperitoneal injection. Moreover, SL-GAC ameliorated significantly the hepatic damage and iron overload in a mouse ALD model induced by alcohol in our previous study, and the mice were intragastrically administered with SL-GAC. In the present study, we conducted experiment with reference to the administration of SL-GAC on mice ALD model, and the selected administration were effective on iron overload-induced liver injury mice model.

At the end of experiment of the study, and after 12 h of food deprivation, the excrement of mice was collected and frozen in liquid nitrogen. Then, all mice were narcotized with lidocaine and killed by cervical dislocation, the blood was collected from each of the mice by cardiac puncture for biochemical analysis. After the clotting of blood samples, serum was separated by centrifugation and stored at −80 °C until analysis. Liver was immediately excised, weighed, snap-frozen in liquid nitrogen, and stored at −80 °C for further analyses. One part of the liver tissues was fixed in 4% neutral formalin for histopathological analysis.

### Measurement of iron indices and serum parameters

Serum samples of mice were prepared by centrifuging the whole blood for 8 min at 2,000 *g* and stored at −80 °C until used for biochemical analysis. The level of ALT, AST and γ-GT was measured by automatic biochemical analyzer according to the manufacturer’s instructions. Serum iron, liver iron and TIBC were measured with the iron and iron binding–capacity kit. Serum iron concentration was determined using the assay based on the generation of an iron-ferrozine complex. Iron concentration in the digested liver sample was measured spectrophotometrically at 535 nm, following reaction with 2 mM bathophenanthroline disulfonic acid. The TS was directly calculated from the serum iron and TIBC, and UIBC was equivalent to the sum of TIBC and serum iron.

### Determination of liver MDA, SOD, GSH, TG and TC levels

Liver samples were dissected out and washed immediately with ice cold saline to remove as much blood as possible. Then, liver was weighed and homogenized at a ratio of 1:9 (w/v) in 0.9% saline solution. The homogenates were centrifuged at 3,000 rpm for 10 min at 4 °C, the supernatant was harvested and the levels of MDA, GSH, SOD, TG and TC were determined using commercial kits according to the manufacturer’s instructions.

### Measurement of ROS production in liver of mice

The smaller liver fragments (3 × 3 × 3 mm) were embedded by optimal cutting temperature embedding agent in the pre cooled cryostat. After frozen, the tissue sections were made, the thickness was about 5 mm, and 5 M dihydroethidium (be configured with PBS) was used at 37 °C. After incubation for 15 min, rinsed slices with PBS for 3 times and observed under confocal laser scanning microscope (magnification, 200×). Fluorescence intensity was observed, each group of three samples, the same exposure intensity and time to take photographs.

### Western blot assays

Mice liver tissue samples were removed from −80 °C and lysed in radioimmunoprecipitation (RIPA) lysis buffer, followed by grinding with tissue lyser at 60 HZ for 2 min at 4 °C and then put on ice for 30 min. The lysates were centrifuged for 15 min at 12,000 rpm at 4 °C. Bicinchoninic acid kit for determination of the protein concentrations of collected supernatant. Next, equivalent amounts of protein samples were separated by 10% sodium dodecyl sulphate polyacrylamide gel electrophoresis (SDS-PAGE), and transferred to polyvinylidene fluoride (PVDF) membranes. Membranes were blocked with 5% non-fat milk for at least 2 h and then incubated with primary antibody at 4 °C overnight, followed by incubation with a horseradish-peroxidase-conjugated secondary antibody at room temperature for 2 h. The protein bands were visualized using enhanced chemoluminescence. The glyceraldehyde-3-phosphate dehydrogenase (GAPDH) antibody was used as a control.

### Measurement of hepatic index

Body weights and liver tissues of mice were measured at the termination of the study. Hepatic index was estimated from the ratio of liver tissue weight to body weight (liver tissue weight/body weight × 100).

### Histopathology and immunohistochemistry assays

Liver specimens from central zone of medial lobe were cut into about 3 mm thick, fixed in 10% neutral buffered formalin for 12 h and then embedded in paraffin. Next, the paraffin block was sliced into 5 μM thick sections, stained with hematoxylin-eosin according to the manufacturer’s instructions, and examined microscopically for histopathological changes. Meantime, immunohistochemistry (IHC) assay for ferritin of sections obtained from 10% paraffin-embedded tissues was performed by the one step polymer-HRP Detection Kit (BioGenex) according to the manufacturer’s protocols. Subsequently, these stained sections were visualized and photographed under inverted microscope (100 × magnification).

### Statistical analysis

All data were presented as means ± SEM. Differences among groups were assessed by using Student’s *t*-test and one-way analysis of variance. *p* < 0.05 was considered to indicate a statistically significant difference. Calculations were performed with the SPSS, Version 26, statistical software package.

## Results

### *In vitro* study

#### Antioxidant activity of SL-GAC

**Figure 1.**
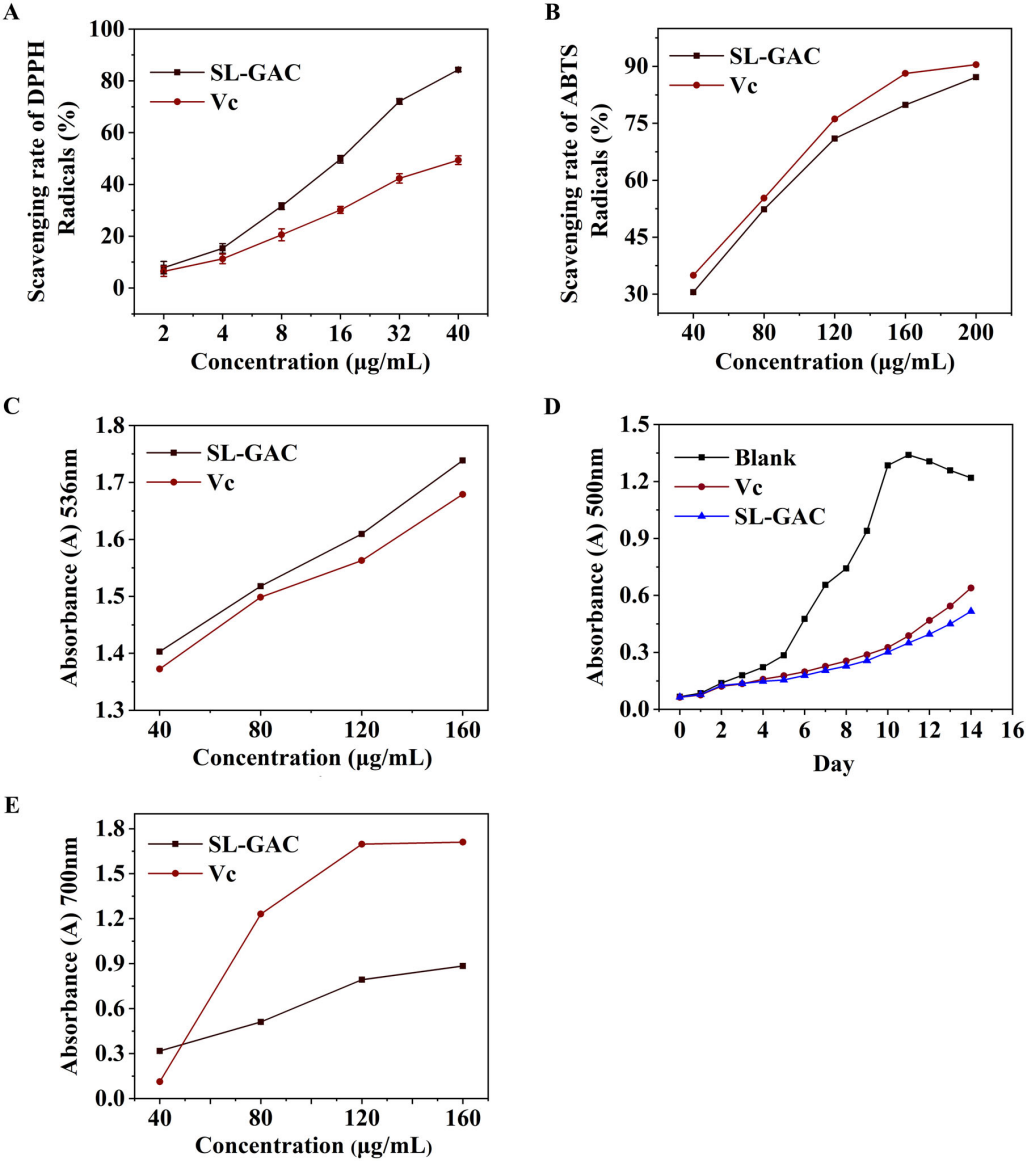
Antioxidant activity and Ferric reducing power of SL-GAC *in vitro*. (A, B) Free radical scavenging activities of SL-GAC and vitamin C (Vc) at different concentrations were determined by 1,1-diphenyl-2-picrylhydrazyl (DPPH) and 2, 2-azinobis (3-ethylbenzo-thiozoline-6-sulfonic acid) (ABTS) radicals scavenging activities, respectively. (C) Inhibition of lipid peroxidation in linoleic acid by the SL-GAC and vitamin C (Vc). (D) Hydroxyl radical scavenging activity of SL-GAC and vitamin C (Vc). (E) Ferric reducing power of SL-GAC and vitamin C (Vc) at different concentrations (40, 80, 120, and 160 μg/mL). Results are expressed as means ± SD (*n* = 3).

For the determination of antioxidant capacity of SL-GAC *in vitro*, DPPH, ABTS and hydroxyl radical scavenging activities, linoleic acid autoxidation inhibition capacity and Fe^3+^ reduction capacity were used. As shown in Figure 1A and B, SL-GAC was identified as the potent antioxidant against DPPH and ABTS with IC_50_ values of 24.92 ± 0.46 µg/mL and 128.36 ± 6.57 µg/mL, respectively compared to vitamin C (IC_50_ = 44.78 ± 0.62 μg/mL and 114.30 ± 0.61 µg/mL). In addition, scavenging activity of SL-GAC on Fenton hydroxyl radicals increased with increase in the concentration of SL-GAC. Excitedly, the ability of SL-GAC to scavenge hydroxyl radicals in Fenton reaction were stronger than those of vitamin C at the same concentrations (Figure 1C). Moreover, the results of inhibition of linoleic acid autoxidation showed that the percentage of inhibition of linoleic acid peroxidation by the SL-GAC was comparable with that of vitamin C during the 14 d of experiment and significantly prolonged the induction period of autoxidation as compared to control (Figure 1D). Furthermore, Furthermore, the reducing capacity of Fe^3+^ showed an accelerating trend with increased concentrations of SL-GAC (Figure 1E). The results, therefore, indicated that SL-GAC appeared to possess potent antioxidant activity and free radical scavenging ability *in vitro*.

### *In vivo* study

#### Iron levels of serum and liver tissue in mice

**Figure 2.**
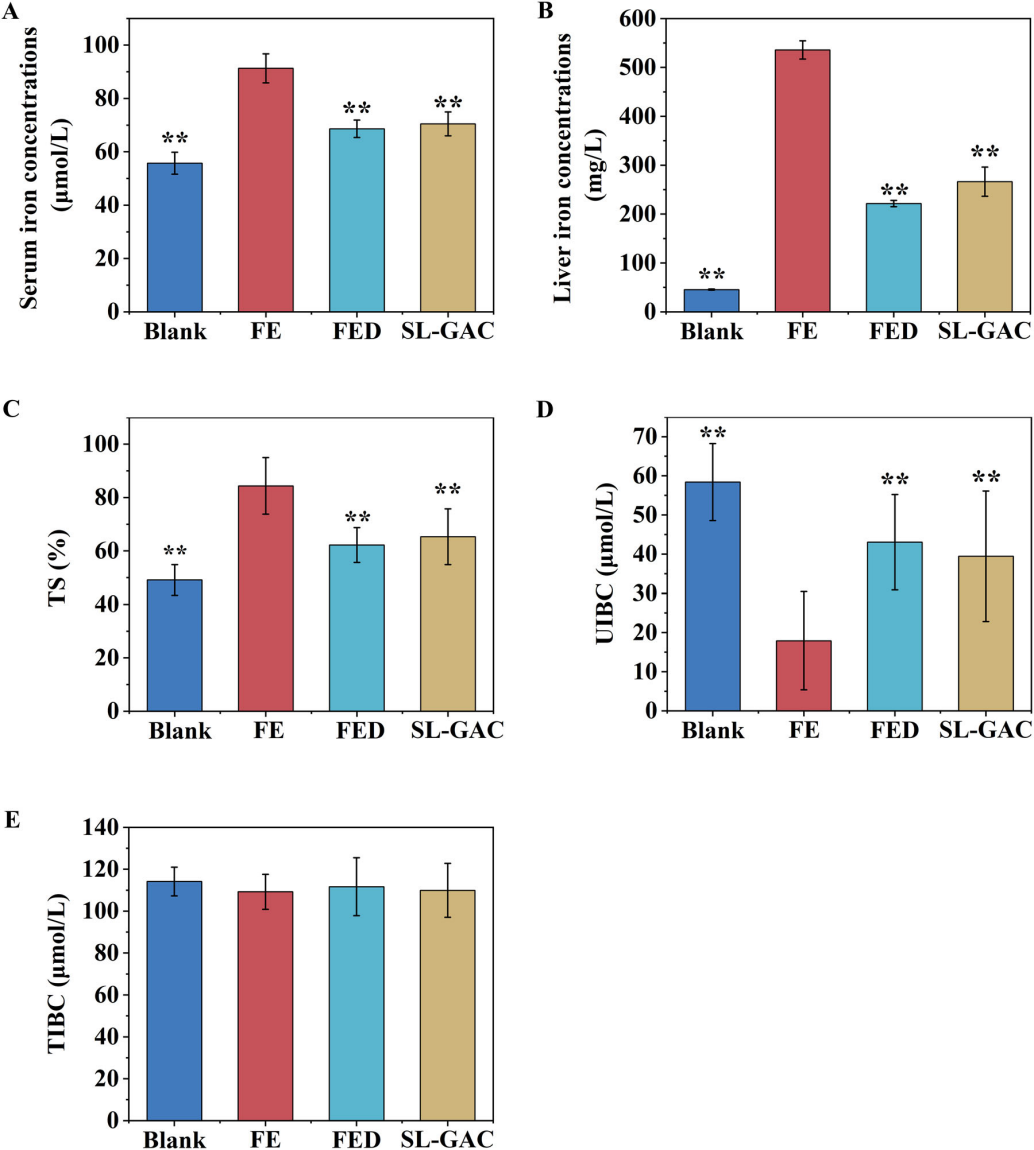
SL-GAC reduces iron accumulation in liver injury mice by iron overload induced. (A, E) Serum iron parameters, including serum iron and total iron binding capacity (TIBC) measured in mice. (B) Hepatic iron concentrations in the liver tissues of C57BL/6J mice in different groups. (C, D) Unsaturated iron-binding capacity (UIBC) and transferrin saturation (TS) were directly calculated from the serum iron and TIBC. Data are reported as mean ± SEM (*n* > 6). *p < 0.05, **p < 0.01 compared with the iron-overloaded group.

Compared to normal mice, the serum iron, hepatic iron and TS levels increased to 63.84%, 1079.31%, and 71.69% in the iron-overloaded mice, respectively. Administration of SL-GAC significantly reduced serum iron content to 29.57%, and liver iron level to 101.16%, and TS level to 29.18% (*p* < 0.01) (Figure 2A, B and C). Additionally, UIBC, a measure of the iron binding reserve of serum is significantly lower in FE group relative to control mice. When the iron overloaded mice were treated with SL-GAC, there was a significant increase in serum UIBC (*p* < 0.01) (Figure 2D). But TIBC levels in the serum were not significantly different among the four groups (Figure 2E). These results revealed that SL-GAC can normalize the liver iron, and the serum iron levels of iron overloaded mice.

#### SL-GAC chelates extra-hepatic iron in mice

**Figure 3.**
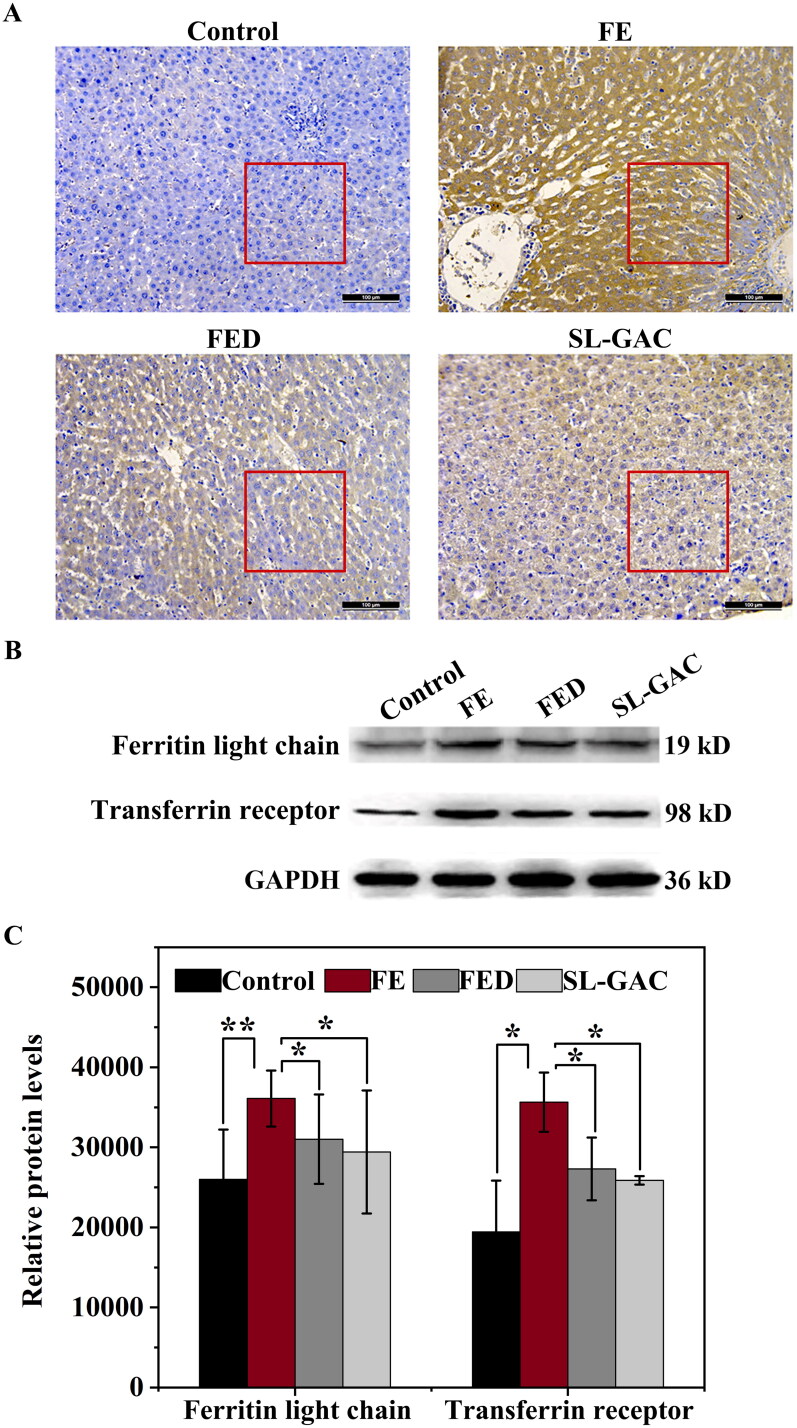
Effects of SL-GAC on protein expression levels involved in iron metabolism. (A) Immunohistochemistry against ferritin in liver tissue samples of mice. (B, C) Protein levels of ferritin light chain and transferrin receptor in the liver of different groups as determined by immunoblotting (*n* > 5). Results were normalized to the internal control GAPDH and presented as relative expression level calculated.

Ferritin is a hepatic protein that plays vital roles in diagnosing and predicting diseases, its levels reflect body iron stores and are elevated with inflammation in chronic liver injury (Cao et al. 2021). Immunohistochemistry evaluation showed that the positive immunohistochemical reaction (brown color) of liver tissue in FE group was higher than that in the control group, while the positive brown staining of liver tissue in SL-GAC group was significantly less compared with FE group (Figure 3A). Furthermore, western blot assays showed that the elevated levels of the ferritin light chain in FE group compared with control group, was remarkably decreased in SL-GAC group (Figure 3B-C). The transferrin receptor (TfR1), a type II transmembrane glycoprotein that binds transferrin (Tf) and mediate cellular iron uptake through endocytosis of iron-loaded transferrin. Inside the cell, Tf is trafficked to early endosomes, delivers iron, and then is subsequently directed to recycling endosomes to be taken back to the cell surface (Mayle et al. 2012; Candelaria et al. 2021). Iron overloaded mice showed significantly increased TfR1 level compared with blank mice, whereas SL-GAC supplementation significantly reduced TfR1 accumulation (Figure 3B-C).

#### Biochemical parameters

**Figure 4.**
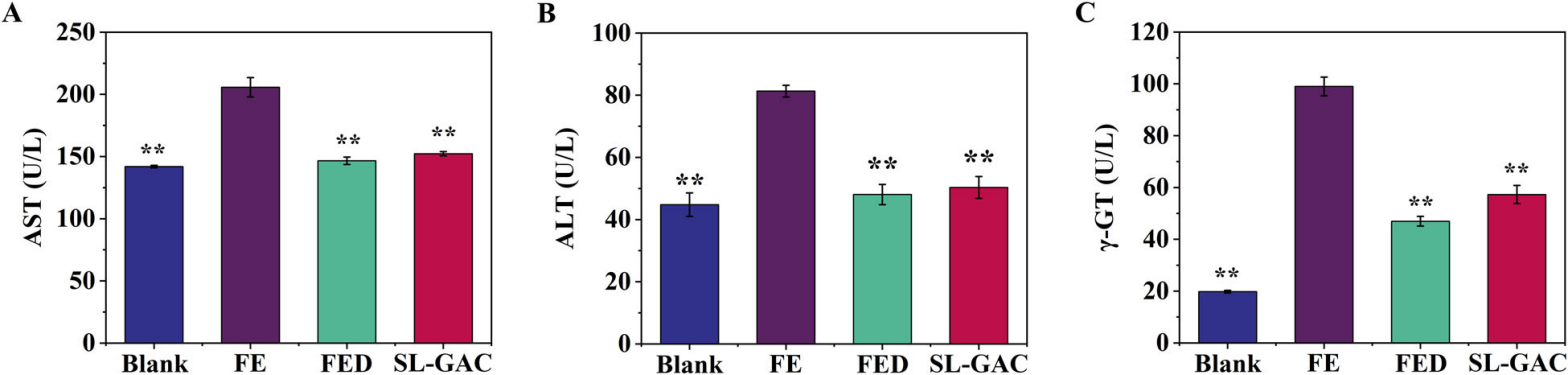
Effects of SL-GAC on serum biochemical indexes. Levels of (A) Aspartate transaminase (AST), (B) Alanine transaminase (ALT) and (C) Gamma glutamyl transferase (γ-GT) in serum samples of mice in the different treatment groups were measured using assay kits. Data represented as means ± SD (*n* > 6), *p < 0.05, **p < 0.01 versus iron-overloaded group.

The ALT, AST and γ-GT are enzymes produced by the liver that indicate liver damage or disease at high concentrations in the blood (Van Beek et al. 2013). Compared to the blank group, levels of these serum markers increased significantly in iron-overloaded mice (*p* < 0.01), interestingly, treatment with SL-GAC reversed the increase of serum ALT, AST and γ-GT that led to reduced liver injury induced by iron overload in mice (*p* < 0.01) (Figure 4A–C).

#### ROS production

**Figure 5.**
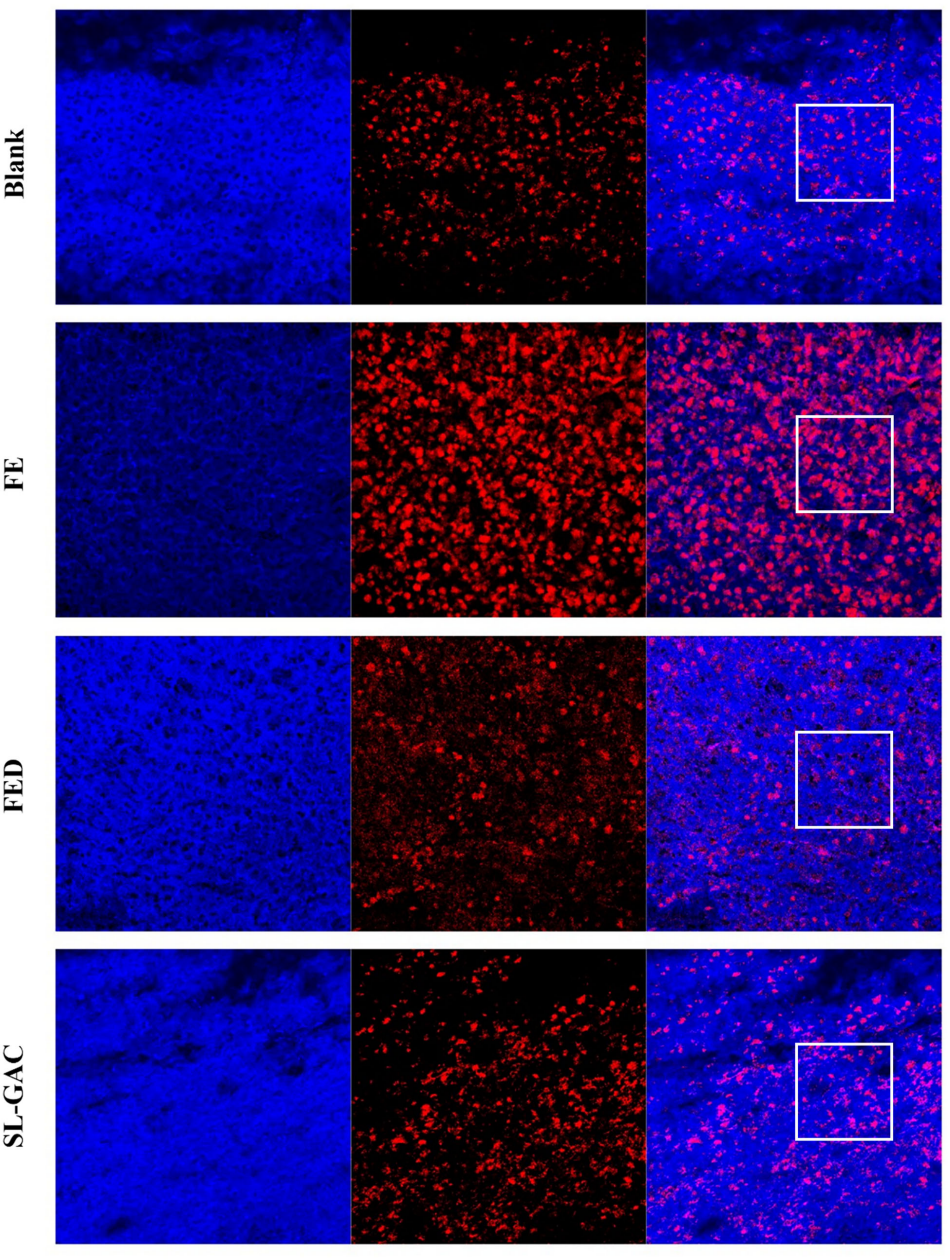
Effects of SL-GAC on ROS level in the liver of mice treated by iron overload.

Oxidative stress is a major pathogenetic event occurring in several liver disorders ranging from metabolic to proliferative ones. The effects of SL-GAC on ROS levels in liver tissue of iron-overloaded mice were detected using the Dihydroethidium staining. As shown in Figure 5, the intensity of the red fluorescence was stronger in liver of iron-overload-induced mice compared with the control group, the increased red fluorescence intensity was attenuated significantly by SL-GAC (Figure 5), indicating that SL-GAC could reduce effectively the excessive ROS production.

#### Oxidative stress of liver tissue

**Figure 6.**
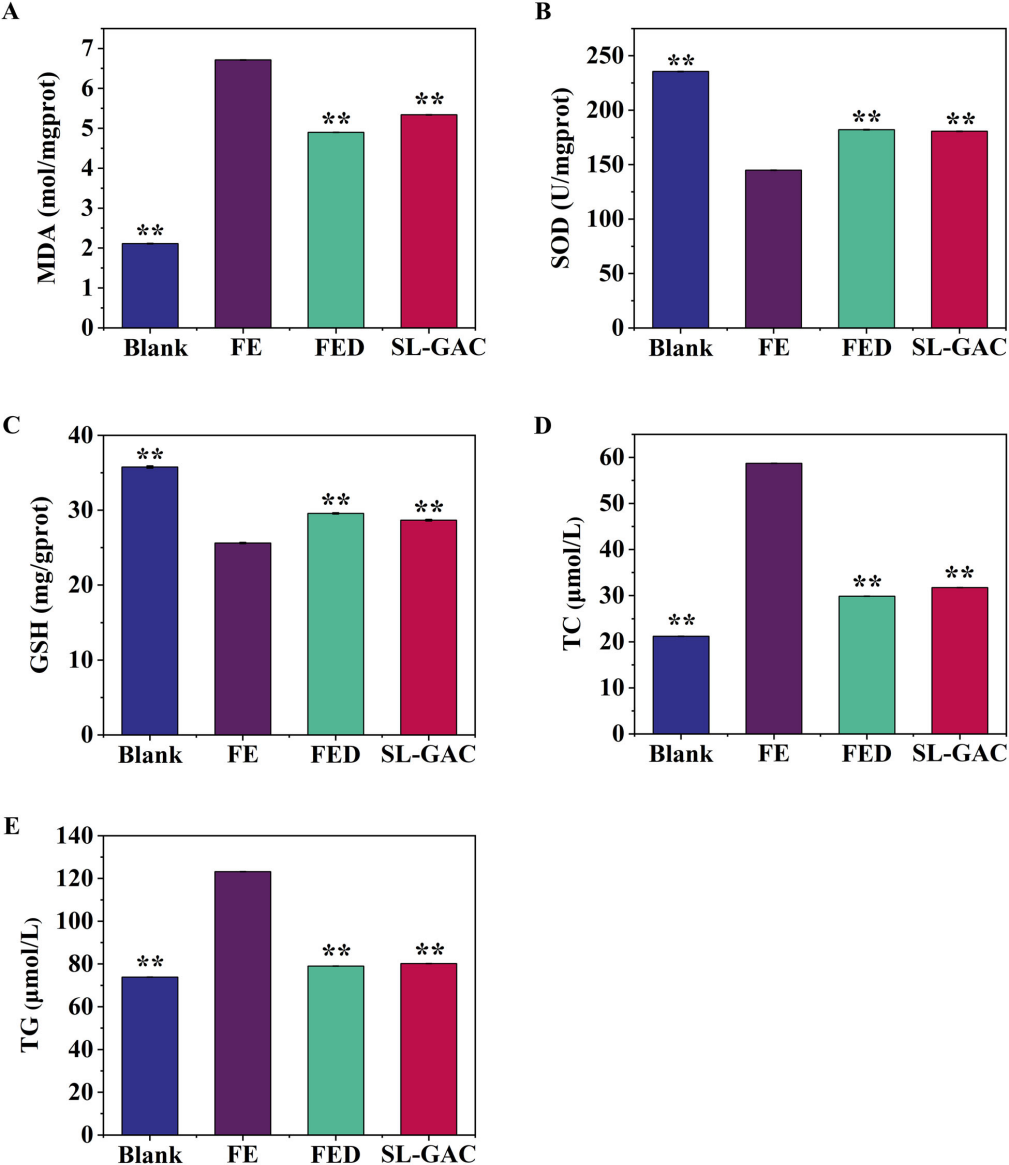
Effects of SL-GAC on hepatic lipid peroxide and antioxidant levels of iron-overloaded mice. (A) Malondialdehyde (MDA); (B) Superoxide dismutase (SOD); (C) Glutathione (GSH); (D) Hepatic total cholesterol (TC); (E) Hepatic triglyceride (TG) levels in iron overload induced mice and effect of treatment with SL-GAC. Data (*n* > 6) was presented as the means ± SD. *p < 0.05, **p < 0.01 compared with the iron-overloaded group.

The levels of SOD, GSH, and lipid peroxidation product MDA were evaluated to assess the level of oxidative damage and the antioxidant effects of SL-GAC. As expected, the iron overloaded mice showed increased MDA while SOD and GSH levels decreased compared with blank mice. Conversely, the model mice supplemented with SL-GAC showed markedly reduced MDA with elevated SOD and GSH levels in comparison to the FE group (*p* < 0.01) (Figure 6A–C). We further elucidated the effects of SL-GAC on hepatic steatosis in iron treated mice. As shown in Figure 6D-E, treatment with iron overload induced a remarkable increase of TC and TG in the liver compared to blank group, and the overexpression of total cholesterol (TC) and triglyceride (TG) in FE group were significantly reversed in the SL-GAC group (*p* < 0.01). These results demonstrated that SL-GAC can effectively attenuate lipid peroxidation and reduce oxidative stress induced by iron-overload in mice.

#### Histopathological liver damage and physiological indexes

**Figure 7.**
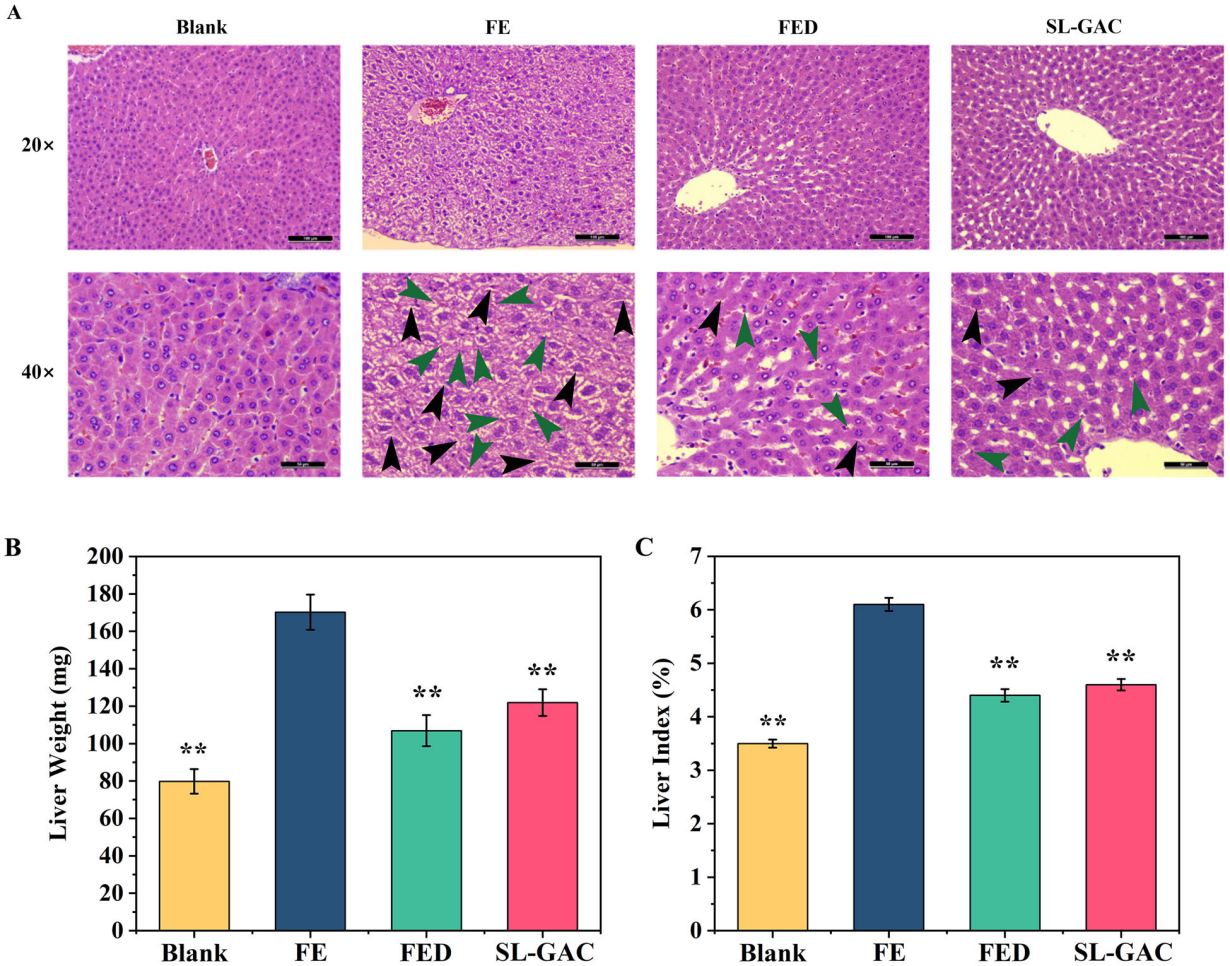
Effects of SL-GAC on histopathological liver damage and physiological indexes of iron overload-induced mice. (A) Representative histologic images of liver tissues of mice obtained by HE staining. (B, C) The liver weight and liver index of mice in different groups were showed. Data represented as means ± SD (*n* > 6), *p < 0.05, **p < 0.01 versus FE group.

Morphometric analysis of liver tissue *via* HE staining revealed that the structure of hepatic lobules was intact in the blank group, as characterized by the radiating arrangement of hepatic cells and cords around the central vein, as well as the hepatic sinusoids were regularly distributed. In FE group, the hepatic lobule structure appeared disintegrated, meanwhile, proliferating fibrous tissues and pseudolobules were seen (black arrows), and scattered lymphocyte infiltration (green arrows) in the liver interstitium were observed in the liver tissues. Photomicrographs of the liver in the SL-GAC group showed a relatively normal hepatic architecture, with a preserved parenchymal structure and occasional infiltration of inflammatory cells (green arrows) (Figure 7A), indicating that SL-GAC suppresses liver injury in iron-overloaded mice by attenuating the morphological abnormalities and pathological injury of the liver induced by iron. Additionally, we found that liver weights were 53.11%, 37.19%, and 28.38% lower in the blank, FED and SL-GAC groups than that in FE group (Figure 7B). Along with that, liver index of the iron overloaded group was significantly higher than blank (*p* < 0.01), and while that of FED and SL-GAC groups showed a significant recovery compared with model group (*p* < 0.01) (Figure 7C). These results suggested that SL-GAC effectively improved pathological injury and physiological indexes of liver tissue of mice with iron overload-induced liver injury.

## Discussion

Iron plays a crucial role in the body, it is important for us to maintain an adequate supply of iron to synthesize oxygen transport proteins, and participates in numerous processes necessary for normal body functions (Wallace 2016). As there is no active mechanism for the excretion of excess iron from the body, imbalance of iron absorption and excretion leads to a progressive accumulation of iron in tissues and organs. Numerous epidemiological studies have reported that accumulation of iron in the organs is toxic and has been linked to a wide variety of life-threatening conditions, such as liver disease, heart problems and diabetes (Jaishankar et al. 2014). The damage is particularly severe to the liver, which is the main site of iron storage in the body (Anderson and Frazer 2005). Free cellular iron can participate in the Fenton reaction and catalyse the conversion of hydrogen peroxide to a more powerful and damaging hydroxyl free radical (Beaufay et al. 2020). A chronic increase of iron in the body can induce overproduction of free radicals, which further causes oxidative stress and leads to the cellular injury and pathologic change (Uttara et al. 2009). Emerging evidence also suggests that oxidative stress, mediated by free radicals and ROS, may have a role in the pathophysiology of iron-induced liver injury (Galaris et al. 2019; Hassani et al. 2020). Gallic acid (GA), a naturally occurring phenolic acid, is well known as a powerful antioxidant and provides efficient protection against oxidative damage (Sanchez-Martin et al. 2022). However, poor bioavailability of GA is shown due to its low lipid solubility that does not allow its penetration into cell membranes, which results in an inability to achieve its desired pharmacological functions (Ahmed et al. 2018). Our previous studies found that SL-GAC, a complex synthesized from GA and soybean lecithin, significantly increased bioavailability of GA, improved pharmacological effects, and had no effects on safety for the organism (Wu et al. 2019). Presently, we designed an iron-overload induced oxidative stress model in mice to further evaluate the effects of SL-GAC on iron-overload induced oxidative stress and liver injury.

Iron is present in the human body and exerts its physiological functions in both the ferric (Fe^3+^) and ferrous (Fe^2+^) states. Because iron converts easily between its ferrous (Fe^2+^) and ferric state (Fe^3+^), many proteins utilize iron as a cofactor to catalyze redox reactions (Gulec et al. 2014). In the presence of oxygen, excess ferrous iron (Fe^2+^) can participate in the Fenton or Fenton‐like reactions to react with H_2_O_2_ and generate one of the most deleterious reactive oxygen species, hydroxyl radical; see reaction (1) (Zhao 2019).

(1) $${\text{Fe}}^{\text{2}+}+{\text{ H}}_{\text{2}}{\text{O}}_{\text{2}}\to {\text{Fe}}^{\text{3}+}+{\text{ HO}}^{\cdot }+{\text{ OH}}^{-}$$

The hydroxyl radical is an extremely reactive free radical formed in biological systems and has been implicated as a highly damaging species in free radical pathology, capable of damaging almost every molecule found in living cells (Wintola and Afolayan 2011). Among the oxygen radicals, the hydroxyl radical is the most reactive and induces severe damage to adjacent biomolecules (Birben et al. 2012). These radicals have the capacity to join the nucleosides in DNA and cause strand breakage, which contributes to carcinogenesis, mutagenesis, and cytotoxicity (Jeong et al. 2010). Due to the abstraction of hydrogen atom from unsaturated fatty acid, these radicals also are considered to be rapid initiators of the lipid peroxidation process (Yamada et al. 2016). Linoleic acid is the most abundant polyunsaturated fatty acids (PUFA) *in vivo*, and susceptible to lipid peroxidation mediated by oxygen free radicals. Currently, lipid peroxidation is considered as the main molecular mechanisms involved in the oxidative damage to cell structures and in the toxicity process that leads to cell death (Zhang et al. 2018). To the best of our knowledge, among of all antioxidant properties, radical scavenging, reducing power and inhibition effect upon linoleic acid autoxidation are most commonly used for the evaluation of antioxidant activity (Islam et al. 2021), including the determination of DPPH and ABTS which are synthetic stable free radicals *in vitro*. Our results showed that SL-GAC may offer resistance against the oxidative stress by scavenging the Fenton hydroxyl radicals, inhibiting peroxidation of linoleic acid, and enhancing ferric reducing power *in vitro* (Figure 1).

Approximately 80% of total body iron is functional, located in haemoglobin, myoglobin and iron-containing enzymes (Hernando et al. 2014), and the remainder is largely stored as ferritin in the liver, which contains mainly the light chain and can store up to 4500 atoms of iron (Anderson and Shah 2013). However, if the storage capacity of ferritin is reached, free iron will accumulate in the cells and can cause cellular and tissue injury (Kohgo et al. 2008). In addition, an additional fraction of free iron is absorbed in the intestinal tract and circulated in the blood (Kohgo et al. 2008). In the circulation, a smaller amount of iron is usually bound to transferrin (Tf), which is central proteins in the regulation of iron metabolism and involved in transporting iron between absorption, utilization, excretion, reclamation and storage areas (Gao G et al. 2019). Transferrin receptor 1 (TfR1) is a classical functional receptor, when serum Tf-bound iron (Fe_2_-Tf) binds to TfR1, Fe_2_-Tf is internalized by endocytosis (Candelaria et al. 2021). Internalized Fe_2_Tf-TfR1 complexes within the endosome release iron when endosomal pH is acidified (Kohgo et al. 2008). The resulting apotransferrin-TfR1 complex is then recycled back to the cell surface for reutilization (Kohgo et al. 2008). The TfR1, as the main cellular importer of iron, delivers iron to cells *via* receptor-mediated endocytosis and allows for transferrin-bound iron uptake in mammalian cells, thereby plays a role in regulating iron homeostasis by balancing iron uptake with intracellular storage and utilization (Candelaria et al. 2021). As expected, the elevated levels of the serum iron, hepatic iron, TS, ferritin, ferritin light chain and TfR1 in iron-overloaded mice were remarkably restrained in SL-GAC treated group (Figures 2 and 3), which indicates that SL-GAC can reduce iron overload in liver and may be administered as an antidote for management of iron overload.

When hepatocytes are injured or inflamed, higher amounts of alanine aminotransferase (ALT) and aspartate aminotransferase (AST) are released into the extracellular compartment, which increases levels of ALT and AST in serum and further reflects the degree of hepatocyte injury to a certain extent (Goorden et al. 2013; Lim 2020). Therefore, the elevation of serum ALT and AST is considered to be an important index for judging the severity of hepatic injury (Han et al. 2011). The γ-GT is a membrane enzyme found in hepatocytes and biliary epithelial cells, it helps the liver metabolize substances, such as drugs, alcohol, and toxins (Rajagopal and Rafi 2005). High levels of γ-GT in the blood may be a sign of liver disease or damage to the bile ducts, as γ-GT may leak into the bloodstream when the liver is damaged (Whitfield 2001; Beek et al. 2013). In this study, the increase of ALT, AST and γ-GT in the iron overloaded-mice was markedly reversed after treatment with SL-GAC (Figure 4).

Excessive iron increases ROS production *via* the Fenton reaction, which causes oxidative damage and even triggers cell death, ultimately causing hepatic injury (Dixon and Stockwell 2014; Wu et al. 2021). Present study found that SL-GAC substantially decreased the expression level of ROS (Figure 5). To combat the excessive free radicals, living cells are armed with a group of intracellular antioxidant enzymes such as SOD and GSH peroxidase, which act as a first line of defences (Nemmiche 2017). SOD, a metalloenzyme present in all oxygen-metabolizing cells, which converts the extremely toxic superoxide radical into potentially less toxic hydrogen peroxide, and is the key line of defense for organisms against the toxicity of reactive oxygen species (Rahal et al. 2009, 2014). The level of SOD activity can be used to evaluate the degree of liver injury (Rahal et al. 2014). GSH, with the enzymes GSH peroxidase and glutathione disulfide reductase, serve to detoxify hydrogen peroxide to water and molecular oxygen and help scavenge free radicals produced in cells, thereby reducing the damage to the cell membrane (Narayanankutty et al. 2019). Free radicals initiate the lipid peroxidation process in an organism (Gaweł et al. 2004). The MDA is one of the most important products of membrane lipid peroxidation in the cells and the final product of lipid oxidation, which can be used to determine the degree of membrane lipid peroxidation and indirectly measure the degree of oxidative damage (Gaweł et al. 2004; Galaris et al. 2019). Liver injury can lead to the transfer of fatty acids to the liver, resulting in an increase of TG content in the liver, while TC reacts to lipid peroxidation in the liver, and the TC level in the body increases (Gong et al. 2008; Zhou et al. 2021). In this study, the elevated levels of MDA, TC and TG was observed in iron overloaded mice. Interestingly, after treatment with SL-GAC, not only significantly reversed these parameters but also the levels of SOD and GSH in iron-overload mice were markedly increased (Figure 6), further suggesting SL-GAC possesses hepatoprotective effects by attenuating oxidative stress in liver.

Liver index is an important indicator to measure hepatic injury in animal experiments, and it has been used to evaluate the degree of experimental liver damage (Zhu et al. 2021). The changes in liver weight can directly reflect hepatic damage (Wang et al. 2019). The present study showed that iron overload caused the increase in the liver weight and liver index in mice, and SL-GAC effectively alleviated the increase in iron overloaded mice. Further, our findings confirmed that SL-GAC can protect the integrity of the structure of liver tissues and alleviate the damage to the liver organ of iron-overloaded mice (Figure 7). These results indicated that SL-GAC can protect liver tissue from iron overload damage.

## Conclusions

**Figure 8.**
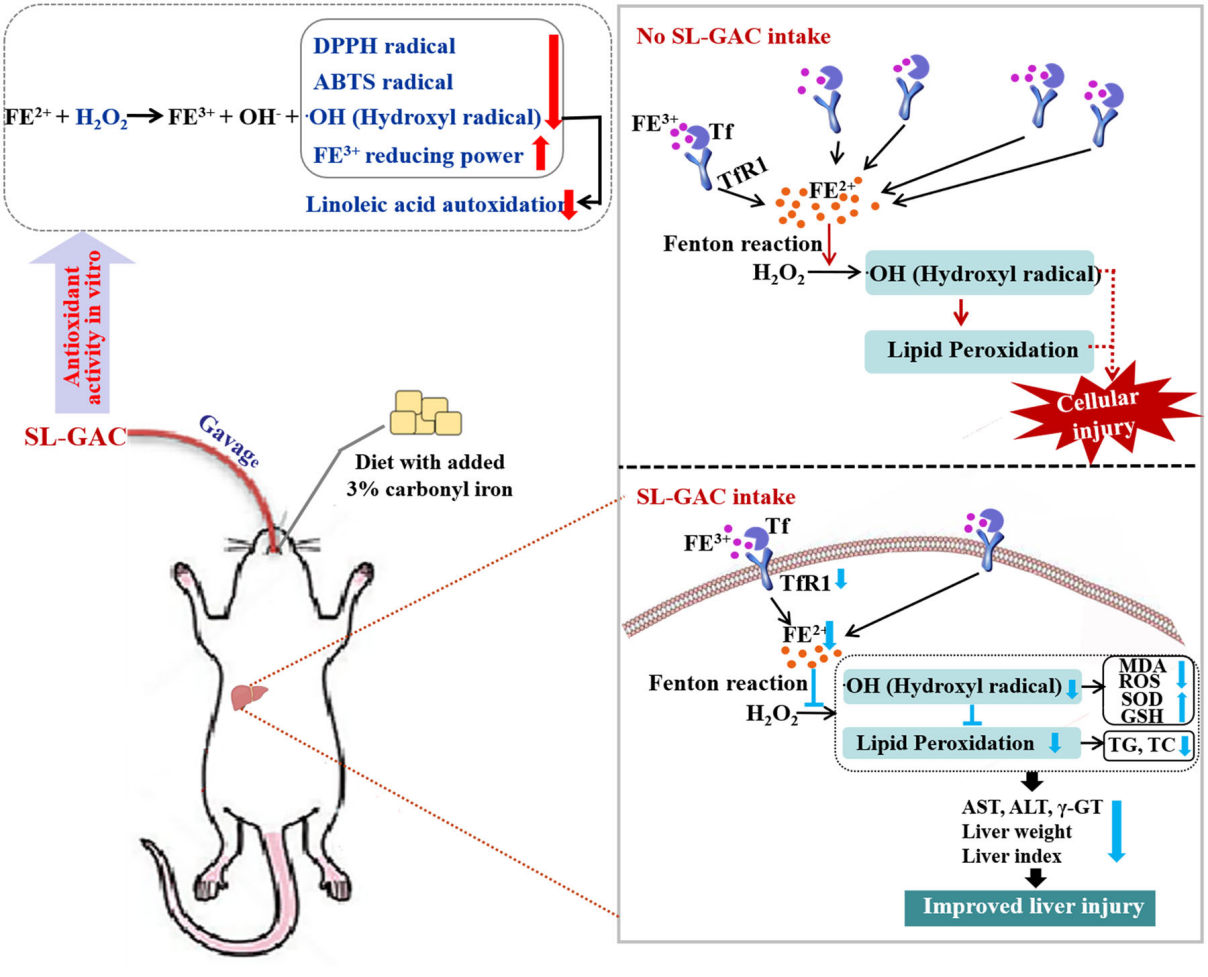
Schematic presentation indicates the suggested mechanism by which SL-GAC-induced hepatoprotective effects. Iron-overloaded mice model is successfully induced by feeding with 3% carbonyl iron-containing diet, followed by intragastric treatment of soybean lecithin with gallic acid (SL-GAC). The SL-GAC significantly inhibits the increase of liver weight and liver index in mice, and as well as effectively prevented the high expressions of liver injury markers (ALT, AST and γ-GT) in iron overloaded mice, which might be a promising therapeutic agent for liver damage. Mechanically, SL-GAC reduces iron accumulation and mitigates oxidative damage through scavenging radical, attenuating lipid peroxidation, restraining ROS production and decreasing levels of TfR1, FL and Ferritin, resulting in the inhibition of oxidative stress to suppresses liver injury in mice with iron overload.

Accumulation of excess iron causes oxidative damage of biomolecules, which causes tissue injury primarily liver dysfunction. Observations from *in vitro* studies concluded that the SL-GAC possesses promising antioxidant capacity and free radical scavenging properties. Further, *in vivo* studies reported the ameliorating effect of SL-GAC against iron-overload-induced liver damage. Treatment with SL-GAC reduces the accumulation of excess iron in serum and liver tissue, restored serum parameters, restrains ROS production and reduces oxidative stress in liver tissue. Further, hepatoprotective effect of SL-GAC were confirmed by physiological indexes and histopathological analyses (Figure 8). Collectively, SL-GAC has great potential as a preventive and therapeutic agent for iron-overload-induced liver injury.
